# Stomach Ulcer as a Rare Cause of Pancreatitis: An Unusual Complication of a Displaced Percutaneous Endoscopic Gastrostomy Tube

**DOI:** 10.7759/cureus.2926

**Published:** 2018-07-05

**Authors:** Saad Saleem, Wissam Bleibel

**Affiliations:** 1 Internal Medicine, Mercy St. Vincent Medical Center, Toledo, USA; 2 Gastroenterology, Mercy St. Vincent Medical Center, Toledo, USA

**Keywords:** percutaneous endoscopic gastrostomy, gastric ulcer, acute pancreatitis, penetration, penetrating injury

## Abstract

A 33-year-old man was admitted to the hospital with upper abdominal pain and melena. Laboratory tests were suggestive of pancreatitis. Computed tomography (CT) of the abdomen showed peripancreatic fat stranding but showed no free air in the peritoneal cavity. Esophagogastroduodenoscopy (EGD) was performed, which revealed an ulcer on the posterior wall of the stomach, caused by the inner tip of the gastrostomy tube, and which had penetrated the pancreas. He had no signs of peritonitis. The gastrostomy tube was exchanged. The patient recovered well with conservative therapy within days.

## Introduction

The gastrostomy tube (G-tube) is the preferred route for enteral feeding in a condition that interferes with a patient's oral intake. It is considered to be a safe procedure. In a prospective study that involved 484 patients, the rate of complications was between 18 percent to 27 percent [1]. We report a rare case of acute pancreatitis resulting from a gastric ulcer penetrating the pancreas, which was caused by the inner tip of a displaced gastrostomy tube. A comprehensive literature review was also performed, which revealed that so far no case has been reported with this association. The purpose of this case is to increase awareness of such potential complications from a displaced percutaneous endoscopic gastrostomy (PEG) tube.

## Case presentation

**Figure 1:**
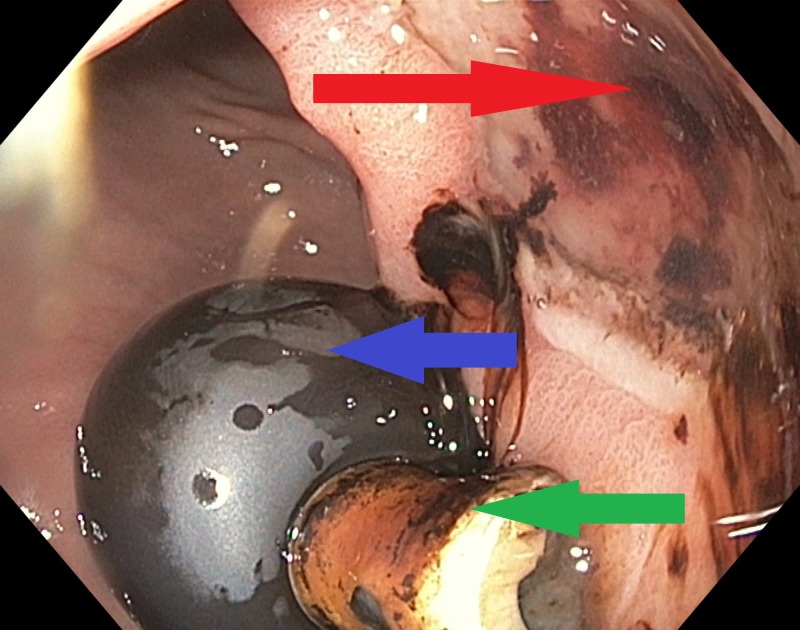
Endoscopic findings are showing active clean base gastric ulcer with no high-risk stigmata (red arrow), also showing balloon (blue arrow) and inner tip (green arrow) of the gastrostomy tube.

A 33-year-old-man who was a nursing home resident with a significant past medical history of anoxic brain injury after a drug overdose presented to the emergency room with upper abdominal pain and black color stools for the last one week. He was refusing tube feedings. He had a PEG tube placed for five years, and it was exchanged one month ago due to malfunction. He was alert to person and place at baseline. However, he was in mild distress due to the abdominal pain. An abdominal examination showed a soft abdomen with mild epigastric tenderness to palpation and the presence of active bowel sounds. A PEG tube was noted in the epigastric area left lateral to the midline. The external bumper of the PEG tube was observed to be more than 10 cm from the skin line. The rectal exam showed melanic stool (guaiac positive). Initial laboratory findings were as follows: amylase: 500 U/L (50 – 150 U/L), lipase: 900 U/L (10 – 140 U/L), hemoglobin: 12.5 g/dL (14 – 18 g/dL), white blood cells (WBC): 4400/µL (3800 – 11000/µL), platelets: 240,000/µL (140,000-400,000/µL), aspartate transaminase (AST): 21 IU/L (10-40 U/L), alanine transaminase (ALT): 23 IU/L (7-56 IU/L), alkaline phosphatase (ALP): 72 IU/L (32-110/IU/L), blood urea nitrogen: 7 mg/dL (6-23 mg/dL), creatinine: 0.6 mg/dL (0.6-1.5 mg/dL), international normalized ratio: 1.04. A computed tomography (CT) scan with intravenous and oral contrast was performed which revealed fat-stranding around the pancreas, suggestive of pancreatitis. An abdominal ultrasonography ruled out any stone or sludge within the gallbladder or bile duct. Fluoroscopy confirmed the position of the gastrostomy tube within the stomach without any extravasations of dye into the peritoneal cavity. Esophagogastroduodenoscopy (EGD) revealed a posterior gastric wall ulcer that was caused by the inner tip of the PEG tube. The ulcer base was deep and clean with no high-risk stigmata (Figure 1). The external bumper of the PEG tube was pushed down to 2 cm from the skin line. The patient was started on proton pump inhibitors (PPI). His pancreatic enzymes continued to trend downward. He was started back on tube feeds, which he tolerated well. Repeat EGD in eight weeks showed a resolution of the gastric ulcer.

## Discussion

Gastrostomy tube placement is a very effective method for long-term (> 4-6 weeks) enteral nutrition. However, complications from the gastrostomy tube placement can be immediate (pneumoperitoneum, ileus, damage to the intraabdominal organ) or delayed, demonstrating after the gastrostomy tract has matured (buried bumper syndrome, and colocutaneous fistula formation). The most severe complications are rare and include peritonitis, necrotizing fasciitis, and aspiration [2]. Studies have revealed that complications are more likely to occur in elderly patients with comorbid illnesses [3].

A review of the literature showed that acute pancreatitis could be precipitated by the internal bumper of the gastrostomy tube migrating from the abdominal wall with resultant obstruction of the ampulla of Vater. Our patient presented with acute pancreatitis due to a posterior gastric wall penetrating (confined perforation) ulcer into the pancreatic parenchyma and this is suspected to be the cause of the acute pancreatitis. We excluded alcoholic, biliary, and medication-induced pancreatitis. To the best of our knowledge, there has been no case report like this in the literature.

In our patient, we assumed that the inner tip of the G-tube had caused the posterior gastric wall ulcer. The mechanism for the posterior gastric wall ulceration leading to penetration is the mechanical damage from the inner tip of a gastrostomy tube. The external bumper of the PEG tube should be a couple of centimeters out from the skin line. When this distance is less, the inner tip of the PEG tube may traumatize the posterior wall of the stomach. In these cases, the external bumper should be adjusted, or the PEG tube would need to be replaced with a non-balloon replacement gastrostomy tube or with a gastrostomy tube in which the inner tip is within the inflated balloon [4]. In our case, the external bumper was adjusted to prevent further trauma to the posterior gastric wall.

Penetration and perforation have the same pathophysiology, except that the ulcer does not erode into the peritoneal cavity. Studies have shown that 20 percent of peptic ulcers can penetrate, but only a small proportion becomes clinically significant [5]. Antral and duodenal ulcers carry the possibility of penetration into the pancreas. Indeed, in our patient, the ulcer was located on the posterior wall of the stomach and had penetrated the pancreas. The patient was treated conservatively and was discharged back to the nursing home on a proton pump inhibitor. A repeat EGD in eight weeks showed the resolution of the gastric ulcer.

## Conclusions

We described an exceptional case of acute pancreatitis caused by the inner tip of a displaced G-tube causing a posterior gastric wall ulcer with penetration into the pancreas. Such a complication has not been reported in the literature. We emphasize that a physician should keep the external bumper of a PEG tube within a couple of centimeters from the skin line and should be vigilant of this rare complication in appropriate settings.
